# Statically foveated freeform OST-HMD system with wide FOV and high perceived resolution

**DOI:** 10.1038/s41377-026-02291-9

**Published:** 2026-05-18

**Authors:** Pengyinjie Lyu, Hong Hua

**Affiliations:** James C. Wyant College of Optical Sciences, The University of Arizona, Tucson, AZ 85721-0094 USA

**Keywords:** Displays, Imaging and sensing

## Abstract

Head-mounted displays (HMDs) based on the well-established rectilinear sampling method are subject to the inherent trade-off between wide field of view (FOV) and high spatial resolution. This challenge limits their broader application due to constraints in manufacturing high-resolution displays and the substantial data bandwidth required for rendering, storage, and transmission. Foveated display technology alleviates this issue by allocating resources differently between the region of interest and the peripheral region. However, most existing solutions rely on dynamic dual-resolution schemes that are costly and complex, requiring multiple displays or optical paths, two-dimensional steering mechanisms, and eye-tracking systems. We propose and demonstrate a perception-driven approach to the design of a three-element freeform eyepiece featuring spatially varying optical power. The novel eyepiece enables the creation of a statically foveated optical see-through HMD, yielding a display of an 80° diagonal FOV and a peak resolution density of 60 pixels per degree with a 4 K display panel. The system offers high perceived resolution across the FOV with imperceptible or minimal degradation and resolution discontinuity with eye movements. Our approach eliminates the need for eye tracking, scanning mechanisms, or multiple displays, significantly reducing hardware complexity. Compared to the rectilinear sampling scheme offering the same peak resolution density and FOV, our system reduces pixel usage by more than 35% or equivalently 4.4 million fewer pixels.

## Abstract (web summary)

A perception-driven freeform eyepiece enables statically-foveated optical see-through head-mounted displays (OST-HMDs) with an 80° FOV and over 60 pixels per degree (PPD) peak resolution, delivering high perceived image quality while reducing pixel demand by over 35%.

## Graphical abstract

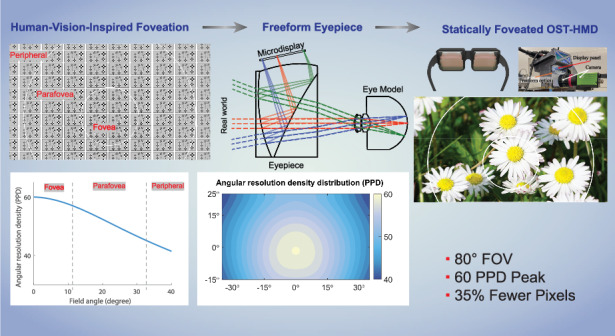

## Introduction

Technological advancements in head-mounted displays (HMDs) have enabled a wide spectrum of applications across various fields^1^, such as medical training^2^, education^3^, and entertainment^4^. Achieving both a wide field of view (FOV) and high angular resolution, however, remains a fundamental challenge. The conventional approach to eyepiece designs for HMD systems adopts the well-established rectilinear sampling scheme that distributes a finite number of pixels uniformly across the FOV. It leads to an inherent trade-off: expanding the FOV reduces angular resolution density while maintaining high resolution limits the achievable FOV. For example, achieving a resolution density of 60 pixels per degree (PPD), equivalent to the visual acuity of 20/20 vision, across a 120° FOV would require display panels nearly 50 million pixels, far beyond the capabilities of current display and computing technologies. Due to the sharp degradation of the visual acuity of the human visual system (HVS) with the increase of eccentricity from the fovea, however, over 95% of the 50 million pixels are wasted from the standpoint of perceived resolution instantaneously.

Inspired by the characteristics of the HVS, various foveation strategies have been explored to mitigate the inherent trade-off between resolution and FOV. These methods aim to identify a user’s region of interest (ROI) and dynamically allocate limited resources, such as pixels or data bandwidth, more efficiently between the ROI and the peripheral visual field. Several dynamically foveated display or imaging systems have been proposed^5–16^, typically featuring a high-resolution foveated region with a narrow FOV and a lower-resolution peripheral region with a relatively broader FOV. These systems often rely on eye-tracking devices or equivalent mechanisms to determine the user’s instantaneous gaze direction and thus identify the current ROI. The high-resolution foveated region is then dynamically steered to align with the gaze direction using either mechanical or optical scanning systems^10–16^. However, these dynamically foveated displays tend to be complex, costly, and bulky because they often require multiple display modules or separate optical paths for multi-resolution rendering, integrated eye-tracking systems, and two-dimensional (2D) steering mechanisms. In the case of optical see-through HMDs (OST-HMD), the integration of dynamic foveation becomes even more challenging due to the need to maintain a see-through optical path along with multiple virtual image paths.

To overcome these limitations, we recently proposed a perception-driven static foveation approach to HMD designs^17,18^. Instead of relying upon complex optical architectures, eye-tracking support, and scanning mechanisms, this method leverages the relatively fixed alignment of HMDs to the user’s eyes and the statistical distribution of human eye and head movements and delivers a wide-FOV virtual display with a spatially varying angular resolution density. It enables the ability to efficiently allocate the limited pixel resources according to the perceptual characteristics of the HVS and eye movement range. Our prior work in^17^ mainly focused on the formulation of static-foveation method, the development of perception-driven performance evaluation framework, the establishment of systematic optimization process, and the experimental validation of the static-foveation method. The prior work in^18^ demonstrated the feasibility of the perception-driven approach through the design and prototype of an immersive HMD featured with custom-designed eyepiece optics of spatially varying optical power. The immersive benchtop prototype, however, is very bulky and heavy, offers relatively low optical performance, and is unable to support optical-see-through capability due to the adoption of a rotationally-symmetric optics architecture.

**Fig. 1:**
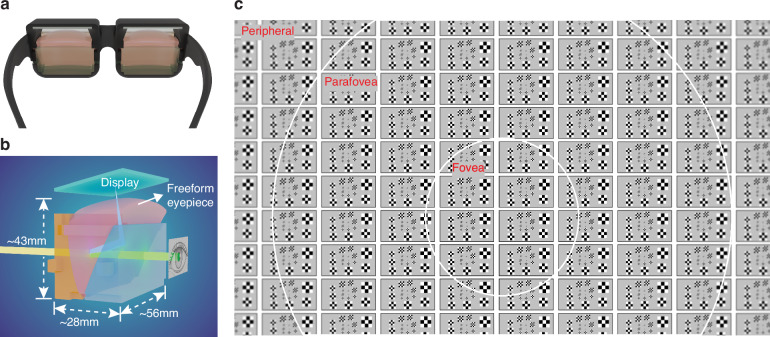
Statically foveated OST-HMD. **a** 3D model of the prototype system. **b** 3D model of a monocular optical system comprising a custom-designed freeform eyepiece and a micro-display. **c** The simulated virtual display after distortion correction via CodeV®

In this paper, we present the design and experimental validation of a statically foveated glass-style OST-HMD system, as shown in Fig. 1a, which yields a virtual display of an 80° diagonal FOV, a peak resolution density of over 60 PPD when combined with a 4 K miniature display of 10.4 μm pixel pitch, and a see-through path of an angular resolution of 0.5 arc minutes. The OST-HMD system is enabled by a novel design and optimization of a three-element freeform eyepiece (Fig. 1b) featuring spatially varying optical power by extending to the perception-driven static foveation method. Compared to the rectilinear sampling scheme offering the same peak resolution density and FOV, our system reduces pixel usage by more than 35% or equivalently 4.4 million fewer pixels and offers a higher perceived angular resolution in regions of frequent eye movement. Compared to dynamically foveated schemes, it not only provides foveation characteristics without the need for eye tracking or complex steering optics but also delivers continuous perception of resolution variation without an abrupt drop of resolution in dual-resolution systems.

## Results

### Statically foveated OST-HMD system

Figure 1a shows the 3D model of our perception-driven statically foveated OST-HMD system which accounts for the characteristics of eye and head movements and the perceived visual effects in the design process. The monocular optical system consists of a custom-designed freeform eyepiece (Fig. 1b) featuring spatially varying optical power and a high-resolution micro-display panel with uniform pixel density. A monocular assembly of the optical system measures about 56 mm, 43 mm and 28 mm in width, height, and depth, respectively, which is comparable to the case of an Apple ® Airpods Pro. The eyepiece was optimized for a 1.87-in. display panel with pixel resolution 3840 × 2460 and a 10.4 µm pixel pitch, yielding a virtual display of an 80° diagonal FOV (±35° horizontal, −15° to +25° vertical) and a peak angular resolution density of over 60 PPD. It uses 35% less pixels than a rectilinearly sampled non-foveated design of the same FOV and resolution density. The virtual-display path is optimized to provide high perceived resolution with a smooth, continuous resolution falloff toward the periphery. Using a mosaic of a modified Briggs target, Fig. 1c shows the optical simulation of the perceived virtual display after distortion correction. Each Briggs target consists of 16 checkerboards varying in the number of pixels per square and the number of squares in a board. The smallest checkerboard contains 2 squares with 1 pixel per square for the Nyquist frequency. The angular resolution maintains at about 60 PPD in the central fovea region (±10°), degrades from 60 to 48 PPD in the parafovea region (±10°–30°), and drops to about 40 PPD at the edge field (40°). The see-through path of the eyepiece is optimized to yield excellent optical performance across a wide FOV. As predicted by optical simulation, it offers an average modulation transfer function (MTF) value exceeding 50% at the frequency of 30 cycles per degree (equivalent to 20/20 vision) across all fields and see-through distortion below 0.1%.

### Freeform eyepiece with spatially varying optical power

**Fig. 2:**
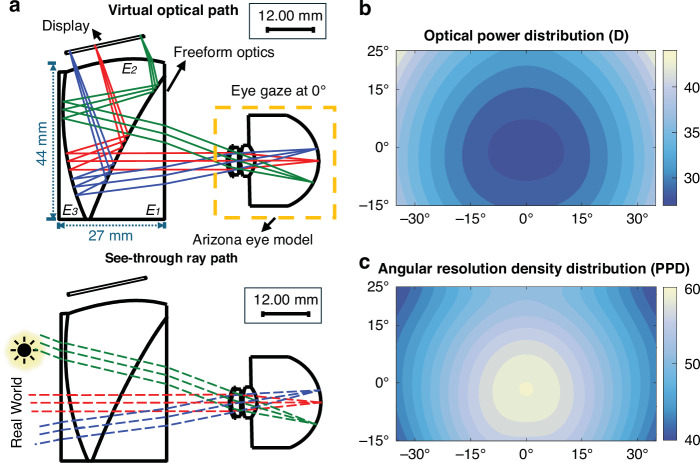
Optical design of a freeform eyepiece with spatially varying optical power. **a** The 2D cross-sectional optical layout of virtual image optical path and see-through optical path of statically foveated freeform OST-HMD with Arizona eye model when eye is gazed at 0°. **b** The optical power distribution of the eyepiece optics in field map. **c** The angular resolution density distribution in field map

The 2D cross-sectional optical layouts of the designed freeform eyepiece, integrated with the Arizona eye model for eye rotations of 0°^19^, are shown in Fig. 2a for the virtual display path and the see-through path, respectively. The eyepiece comprises three freeform elements, where the virtual display path is made of elements E1 and E2 and the see-through path is composed of the sandwich of all three elements. Overall, the optical surfaces in the virtual display path are optimized to deliver a spatially varying optical power distribution such that the pixels of the micro-display are allocated non-uniformly across the FOV to offer a targeted angular resolution density distribution and approximate the foveation characteristics of the HVS. The outer surface of element E3 is optimized along with other optical surfaces in the see-through path to correct the optical distortions induced to the see-through rays by the freeform surfaces of elements E1 and E2. The three cemented elements together approximate a plane-parallel plate (without vision correction), leading to negligible distortion and chromatic effects. For simplicity, the remaining paper refers to the optics for the virtual display path as the freeform eyepiece. While the optical design and optimization strategies and the perceived performance of the eyepiece are provided in the section of Materials and methods, this sub-section highlights the key characteristics of the designed eyepiece.

Unlike a conventional eyepiece, the optical power distribution of the freeform eyepiece, ${\varPhi }_{{EP}}$, which is the reciprocal of the eyepiece focal length ${f}_{{EP}}$, varies with the angular distance of a field position $\theta$ from the display center. For a planar virtual image plane, the effective field-dependent focal length, ${f}_{{EP}}\left(\theta \right)$, at a given field angle θ is related to the chief-ray intercept ${h}_{c}\left(\theta \right)$ as ${{f}_{{EP}}\left(\theta \right)=h}_{c}\left(\theta \right)/\tan \left(\theta \right)$, where ${h}_{c}\left(\theta \right)$ is obtained through ray tracing in CodeV® across the entire FOV at an increment of 0.1°. Figure 2b plots the resulted optical power distribution of the eyepiece measured in diopters across the FOV. The optical power of the eyepiece gradually increases from 27.7 diopters at the display center to 43.5 diopters at the 40° diagonal field position.

Owing to the spatially varying optical power distribution, the eyepiece yields spatially varying optical magnification to the uniformly spaced pixels on the micro-display, leading to a foveated virtual display with gradually decreasing angular resolution density. The spatially varying angular resolution density function rendered by the eyepiece, ${F}_{{FD}}$, measured in PPD, is defined as the reciprocal of the angular resolution in degree subtended per pixel. It is a function of the field-dependent effective focal length distribution ${f}_{{EP}}\left(\theta \right)$ and its derivative ${f}_{{EP}}^{{\prime} }\left(\theta \right)$, expressed as

1 $${F}_{{FD}}(\theta )=\frac{1}{{\rm{atan}}(\frac{{p}_{0}}{{f}_{{EP}}\left(\theta \right)/{\cos }^{2}\left(\theta \right)+{f}_{{EP}}^{{\prime} }\left(\theta \right)* \tan \left(\theta \right)})}$$

where ${p}_{0}$ is the pixel pitch of a uniform-resolution display. Figure 2c plots the angular resolution density distribution across the FOV based on a display with a pixel pitch of 10.4 µm. The eyepiece yielded a peak angular resolution exceeding 60 PPD in the central field region. The angular resolution distribution gradually decreases with increasing field angle and reaches about 40 PPD in the peripheral region. It exhibits strong rotational symmetry despite the non-symmetric optical design and approximates the optimal foveated resolution distribution for a statically foveated display based on our previous work^17^, characterized by:

2 $$\bar{{F}_{{FD}}}(\theta )=\left\{\begin{array}{l}{F}_{{peak}}\,\,\,\,\,\,\,\,\,\,\,\,\,\,\,\,\,\,\,\,\,\,\,\,\,\,\,\,\left|\theta \right|\le 1{0}^{\circ }\\ {F}_{{peak}}\times {e}^{-\frac{1}{2}{\left(\frac{\theta -10}{12.89}\right)}^{2}}\,\,\,\,\,\,\,1{0}^{\circ } < \left|\theta \right|\le 3{0}^{\circ }\\ {F}_{{peak}}\times \frac{2.3}{2.3+(\theta -24.63)}\,\left|\theta \right| > 3{0}^{\circ }\end{array}\right.$$

where ${F}_{{peak}}$ is the peak resolution density of the foveated display and is 60 PPD for the freeform eyepiece design.

Without relying upon complex optical architecture or eye-tracking technology, the statically foveated OST-HMD design offers not only excellent perceived optical performance but also significantly improved data efficiency by efficiently allocating the finite number of pixels across the FOV according to the characteristics of the HVS. To demonstrate these benefits, we compared three different methods for eyepiece optical design—the proposed foveated eyepiece with spatially varying optical power, a rectilinear sampled eyepiece with a constant optical power, and a conventional eyepiece with 30% of barrel distortion—in terms of optical power distribution, number of required pixels, and angular resolution distribution. The three eyepiece designs assume the use of displays with the same pixel pitch for a rotationally symmetric FOV of 80°. While the foveated eyepiece and the constant-power eyepiece assume the same angular resolution density of 60 PPD at the center field, the eyepiece with 30% barrel distortion has to compromise to a slightly lower angular resolution of 57 PPD at the center to ensure the same number of total pixel budget as that of the foveated design and maintain at least 10 PPD at the edge field.

**Fig. 3:**
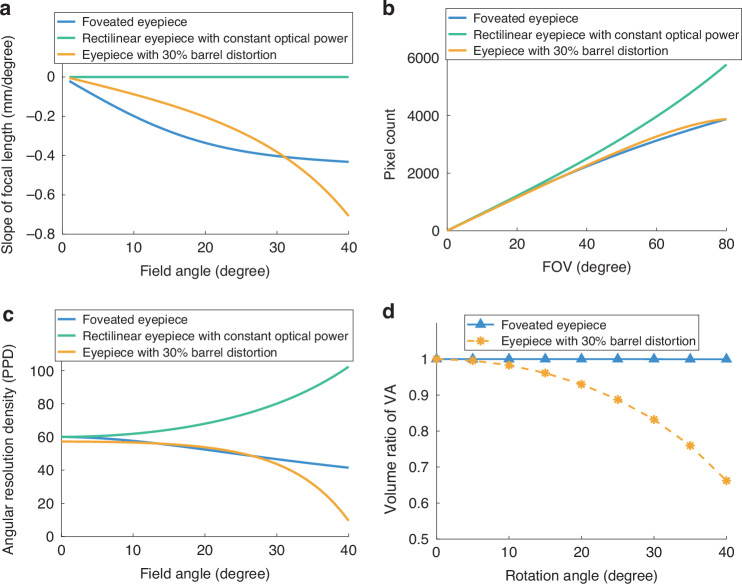
Comparison of optical properties among the foveated eyepiece, rectilinear eyepiece with constant optical power, and eyepiece with 30% barrel distortion. **a** The slopes of focal length across FOV. **b** The pixel utility as a function of FOV. **c** The angular resolution density as a function of field angle. **d** The volume ratio of the perceived resolution as a function of eye rotation angles

Figure 3a compares the slopes of focal length distribution as a function of field angle $\theta$ among the three eyepiece designs. For the eyepiece with constant optical power, the slope is zero. In contrast, the slopes of the foveated eyepiece and the eyepiece with 30% barrel distortion decrease continuously with increasing field angle. Within the central ±30° fields, both designs exhibit slow and moderate decrease of focal length, indicating a smooth and gradual increase of optical magnification with field angles. Beyond this range, however, the slope of the foveated eyepiece levels off while the slope of the eyepiece with 30% barrel-distortion continues to decline rapidly, causing the focal length to drop excessively and substantially reduced angular resolution in the periphery.

Figure 3b plots the required total pixel count for a total FOV up to 80°. Compared to the eyepiece with a constant optical power, the foveated design utilized approximately 14 million fewer pixels for 80° circular FOV. For equivalent pixel budgets, the rectilinear method supports only approximately 59° FOV. By introducing about 30% barrel distortion into the optical system, the conventional eyepiece can extend to an 80° FOV while maintaining the same total pixel count as the foveated design. In terms of data sampling efficiency, both the foveated display and the eyepiece with 30% barrel distortion reduced over 55% of pixel demand to cover the 80° circular FOV with similar peak resolution. The pixel utility across the FOV differs significantly among the three designs. Here, relative pixel utility is defined as the percentage of pixels allocated to the fovea, parafovea, and peripheral regions relative to the total pixel count. Within the fovea (±10° field region), the eyepiece with constant optical power achieves a pixel utility of only 4.4%. The eyepiece with 30% barrel distortion increases the utility to 8.7% and the foveated eyepiece provides the highest utility of 9.2%, indicating that the foveated eyepiece allocates more pixels to the region where eye movements most frequently occur. The pixel utility in the parafovea (±(10°–30°) region) is 56%, 62.4%, and 42.9% for the foveated eyepiece, the eyepiece with 30% barrel distortion, and the eyepiece with constant optical power, respectively. Similarly, the pixel utility in the periphery (±(30°–40° region) is 34.8%, 28.9%, and 52.6% for the three eyepiece designs, respectively. Overall, the eyepiece with constant optical power distributes pixels inefficiently, leading to excessive allocation of pixels to the periphery where the HVS has low visual acuity; while the eyepiece with barrel distortion over-allocates pixels to the parafovea (62.4%), leaving only 28.9% for the periphery and causing severe resolution degradation in the outer field. The foveated eyepiece distributes pixels more effectively, with 56.0% in the parafovea and 34.8% in the periphery, which not only ensures relatively high perceived resolution in the parafoveal region but also preserves sufficient resolution in the periphery to support natural eye movements.

Figure 3c compares the angular resolution density distributions along a radial direction for the three eyepiece designs. The foveated display maintains high angular resolution density in regions associated with frequent eye movements (±15°) and gradually decreases toward the periphery, aligning with the perceptual characteristics of the HVS. In contrast, the eyepiece design with constant optical power exhibits increasing angular resolution density with field angle, opposite to the HVS, which is due to the combined effects of increasing projection angle and viewing distance under a constant focal length. This scheme demands 14 million more pixels to cover the same circular FOV, leading to inefficient pixel allocation and unnecessary pixel demands that do not improve the perceived quality of the display. The eyepiece design with a 30% barrel distortion, to cover the same FOV with the same number of pixels as the foveated eyepiece, necessarily compromised the angular resolution density, approximately 57 PPD, which is 5% lower than that of the foveated design. More importantly, the resolution density drops sharply beyond 20° field, even approaching zero in the peripheral region. Such an abrupt change in resolution is not well aligned with the characteristics of the HVS, particularly in large-FOV systems, and may result in poor perceived performance for large eye rotation angle.

Figure 3d further compared the perceived performance between the foveated design and the eyepiece with 30% barrel distortion, as a function of eye rotation angle up to 35° by applying the metrics proposed in ref. ^17^. For a given eye gaze direction, the perceived resolution of a field angle is determined by obtaining the smaller value between the display resolution distribution and the visual acuity (VA) curve of a 20/20 standard observer. The volume ratio is one of the summative metrics in ref. ^17^ that evaluates whether the perceived resolution of a display is below the perceptible limit of the HVS. It is obtained by computing the ratio of the volume enclosed by the perceived resolution curve of a display across its FOV to the volume enclosed by the VA curve of a 20/20 standard observer. A volume ratio of 1 indicates that the display performs to the limit of the HVS across its entire FOV and a ratio less than 1 indicates the display underperforms to the HVS limit at some field angles. Across the range of eye rotations, the volume ratio of the foveated display remains nearly 100% while the eyepiece with barrel distortion shows a rapid decline in peak perceived resolution beyond 25° of eye rotation and its VA volume ratio is significantly lower than that of the foveated display across the entire sampled angular range. These results demonstrate the substantial advantages of the foveated display in terms of the perceived performance.

### Prototype and performance validation

**Fig. 4:**
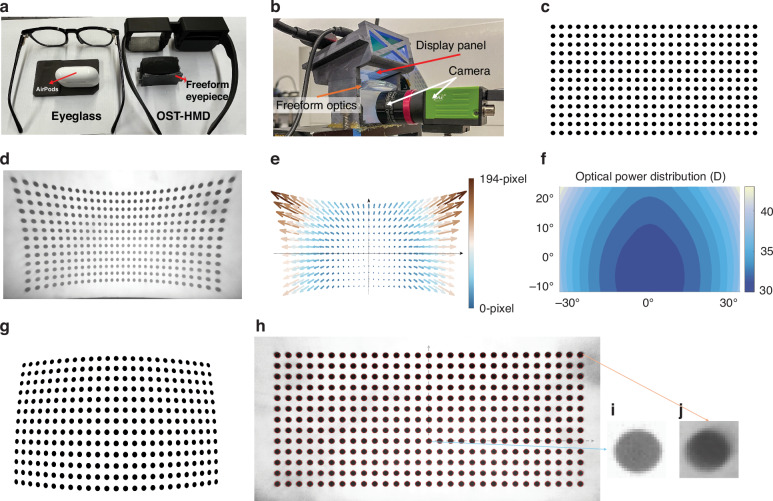
Prototype and calibration of eyepiece optical power distribution. **a** 3D-printed glass-form prototype assembled with the prisms (right). **b** Benchtop prototype of the statically foveated freeform OST-HMD system with a 2 K camera. **c** Original calibration pattern of uniformly sized circles. **d** Image captured through the foveated optics after removing the inherent distortion of the camera system with the original calibration pattern. **e** The optical magnification distribution map across the full FOV. **f** Spatially varying optical power distribution across the full FOV. **g** Prewrapped test pattern generated based on magnification distribution map. **h** Captured image after passing through the foveated optics, corrected for camera distortion, with red dashed circles indicating the original pattern. **i** Zoomed-in view of a circle at the center region. **j** Zoomed-in view of a circle in the top-right peripheral region

To experimentally validate the performance of the statically foveated OST-HMD, we fabricated the custom-designed freeform prisms (shown in Fig. 2a) and assembled a 3D-printed glass-form prototype and a testing prototype as depicted in Fig. 4a, b, respectively. The resulting binocular OST-HMD features a form factor comparable to eyeglasses, while the freeform prism eyepiece is compact, with dimensions similar to an Apple® AirPods case. The experimental prototype adopted a commercially available display panel. Although the panel supports an 80° diagonal FOV, it had a pixel pitch of 24 μm, substantially larger than the 10.4 μm pixel pitch for which the eyepiece was originally designed. This resulted in a peak angular resolution of approximately 26 PPD in the prototype. A pre-calibrated camera with a 2 K sensor and a 3.6-mm imaging lens was positioned at the nominal eye location to capture displayed images through the optical system. The captured images are used to qualitatively validate the spatially varying resolution distribution and optical magnification behavior, rather than to provide absolute image quality metrics. Calibration was performed to obtain the optical magnification distribution of the freeform eyepiece with a spatially varying optical power. The calibration results are utilized to generate a prewrapped input image to account for the optical magnification variation over the FOV such that the resulting output image would appear being uniformly magnified after passing through the foveated optical system. As an example, Fig. 4c shows the original test pattern, composed of a regular array of black dots with equal radius and spacing between adjacent dots, and Fig. 4d shows the resulted image through the foveated eyepiece where the camera’s inherent distortion was removed from the original image captured by the camera. The optical magnification distribution over fields was calculated by comparing the coordinates of the input pattern with those of the captured image, and the resulting distribution was shown in Fig. 4e as a vector map in terms of pixels. Based on this distribution, the spatially varying optical power distribution map across the full FOV was derived, as shown in Fig. 4f. The variation in optical power as a function of field angle shows good agreement with the simulation results. Since the achievable angular resolution scales with the display pixel pitch when the eyepiece provides sufficient MTF, a panel with the targeted pixel pitch of 10.4 μm would proportionally increase the peak angular resolution toward the simulated value of 60 PPD.

Based on the optical magnification distribution in Fig. 4e, the original test pattern shown in Fig. 4c was prewrapped and displayed on the panel, as shown in Fig. 4g. In this prewrapped image, the radius of each dot and the spacing between them decrease from the center to the edge, and the circular shapes are distorted to ellipses. Figure 4h shows the resulted image captured through the optical system, after correcting the camera’s intrinsic distortion. The output closely resembles the original pattern, indicated as the red dashed circles, recovering nearly ideal circular shapes with consistent radii and spacing. Fig. 4i, j show the zoomed-in views of two individual circles from the central and top-right peripheral regions, respectively. The central circle appears sharper and more clearly defined, while the peripheral circle is noticeably blurrier, although it maintains the correct shape and size. These results confirm the accuracy of the calibration method and demonstrate the intended resolution degradation from the center to the periphery in the optical design.

**Fig. 5:**
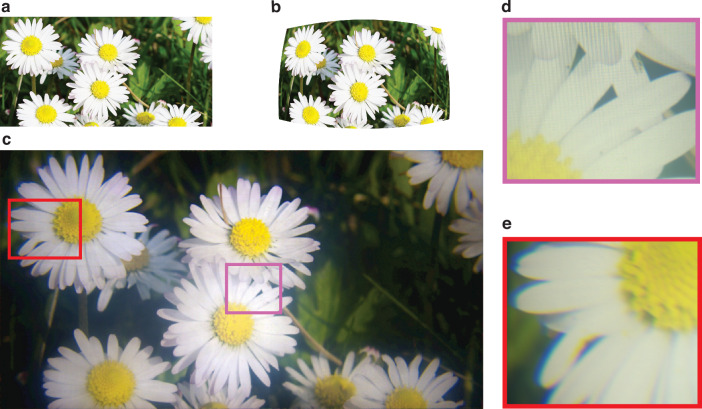
Experimental results. **a** Original undistorted real scene image used as a reference. **b** Prewrapped image as the input source. **c** Image captured through the foveated optics with 3.6-mm focal length camera lens. **e** Zoomed-in view of the central region with 50-mm focal length camera lens. **f** Zoomed-in view of the peripheral region with 50-mm focal length camera lens

Using the same rendering and image capture process described above, a real-scene image was prewrapped with the calibrated magnification distribution, as shown in Fig. 5a, b, respectively. After passing through the optical system and being captured by a 3.6 mm focal length camera lens to cover the full FOV, the resulting image was restored to its original undistorted form while exhibiting a spatially varying resolution distribution, as shown in Fig. 5c. The zoomed-in views of the central and peripheral regions captured using a 50 mm focal length camera lens are presented in Fig. 4e and 4f, respectively. The resolution degradation toward the periphery is clearly observable in these zoomed views, particularly along the edges of the petals.

## Discussion

The statically foveated OST-HMD design proposed in this study offers a compact and efficient solution for mitigating the inherent trade-off between FOV and resolution by utilizing a single, relatively low-resolution display panel without requiring complex optical architectures or demanding eye-tracking or scanning mechanisms. By precisely controlling the optical power distribution of an eyepiece design, we demonstrated a statically foveated display with an 80° diagonal FOV and a peak angular resolution density of 60 PPD by using a 4 K display panel of 10.4 μm pixel pitch. The foveated display resulted in nearly imperceptible resolution degradation in regions of frequent eye movements. A conventional non-foveated design without distortion would require 4.4 million more pixels to offer the same PPD in the center field. To maintain the same amount of pixel demands, a conventional eyepiece design with over 30% distortion is required at the cost of a lower angular resolution in the fovea region and rapid degradation of resolution in the parafovea-peripheral region, leading to noticeably lower perceived resolution across the FOV.

The reduced peak angular resolution of 26 PPD through the prototype is primarily attributable to the limited pixel density of the commercially available display panel used in the experiment. To verify that this reduction is not caused by manufacturing or alignment errors in the freeform prisms, we performed a tolerance analysis based on the fabrication tolerances provided by the manufacturer. Realistic manufacturing and assembly errors were modeled using Monte Carlo simulations incorporating random freeform surface figure perturbations, thickness and alignment variations, and material property deviations within the specified tolerance ranges. A total of 5000 Monte Carlo trials were conducted to evaluate the stability of the virtual image optical path. The results show that, at a 97.7% confidence level, the system maintains an MTF above 0.1 at the Nyquist frequency across all sampled fields, indicating that no severe degradation occurs under realistic fabrication and alignment errors. This confirms that the freeform eyepiece design is robust and that the spatially varying optical power distribution is preserved within the expected manufacturing tolerances. Consistent with this analysis, the experimentally measured optical magnification distribution of the fabricated prototype closely matches the simulated trend, further confirming correct fabrication of the freeform prisms. Under these conditions, the achievable angular resolution is predominantly limited by the display pixel pitch. As angular resolution scales linearly with pixel density when the eyepiece provides adequate optical performance, the reduction from the simulated 60 PPD to the measured ~26 PPD is consistent with the use of a lower-PPI display panel rather than optical fabrication errors.

Future work may explore the use of higher-refractive-index materials to further expand the FOV, reduce the thickness of the eyepiece, and enhance optical performance. Additionally, adopting display panels with higher pixel density could further improve the angular resolution in the foveated region. The data-saving advantages of this approach are expected to become even more pronounced in designs targeting larger FOVs. Finally, an ongoing study further extends to comprehensive user-centered assessments of perceived resolution and visual comfort under representative gaze-direction distributions and usage scenarios.

## Material and methods

### Design and optimization of freeform optics with spatially varying resolution

Freeform surfaces provide greater design flexibility and superior optical performance than traditional spherical or aspherical surfaces due to their additional degrees of freedom for aberration correction. However, practical implementation must account for manufacturing complexity and cost. To balance performance and manufacturability, the optical system in this study is designed to be symmetric about the YOZ plane, reflecting the natural horizontal symmetry of the human head. As shown in Fig. 2a, the freeform eyepiece is composed of three cemented freeform elements with two independent optical paths—the virtual display path and the see-through path. The two elements (E1 and E2) adjacent to the eye model form the main eyepiece for the display path and the last element (E3) serves as a compensation lens for the see-through path when combined with the first two elements. The display is positioned above the eyebrow, with the light path folded inside the main prism E2 through two reflections. All optical surfaces are decentered and tilted relative to the global coordinate system centered with the exit pupil of the system, coinciding with the entrance pupil of the eye model. The system specifications are summarized in Table 1.

**Table 1:** The overall specifications of the system

	Parameters	Specifications
Display	Active panel size	39.94 × 25.58 mm
	Active pixel resolution	3840 × 2460 pixels
	Pixel pitch	10.4 µm
	Exit pupil diameter	10 mm
	Eye relief	≥15 mm
	Material of freeform prism	COP
Optical system	Wavelength	480–625 nm
	FOV	80° diagonally, ~70° (H) * ~40° (V)
	Vignetting	No
	Image quality	MTF > 20% at 60 PPD

The main design challenge lies in optimizing the virtual display path which requires spatially varying optical power distribution to achieve a target angular resolution density distribution, while optimizing the optical performance across the entire FOV. The first step of the design process is to determine the target angular resolution density distribution of a statically foveated display, which is obtained through a complex optimization process by accounting for the target display specifications and requirements, available display resources, the characteristics of eye and head motion, the perceived performance and overall efficiency^17^. The function depicted in Eq. (2) was utilized as the target resolution density distribution, from which the target image height distribution, $\bar{{h}_{c}}\left(\theta \right),$ is iteratively computed across the FOV as follows. For a small field increment $\Delta \theta$, the target image height for the field angle $\theta +\Delta \theta$ is written as $\bar{{h}_{c}}\left(\theta +\Delta \theta \right)=\bar{{h}_{c}}\left(\theta \right)+\bar{\Delta {h}_{c}}\left(\theta \right)$, where $\bar{\varDelta {h}_{c}}\left(\theta \right)$ is the image height increment and can be obtained from the angular resolution density of the virtual display as

3 $$\bar{\varDelta {h}_{c}}\left(\theta \right)=\bar{{F}_{{FD}}}(\theta )* {p}_{0}* \Delta \theta$$

where ${p}_{0}$ is the pixel pitch of the micro-display. The number of pixels covering this angular increment is approximately $\Delta {h}_{c}\left(\theta \right)/{p}_{0}$. The target focal length distribution, $\bar{{f}_{{\rm{EP}}}}(\theta )$, is then computed by applying $\bar{{f}_{{EP}}}\left(\theta \right)=\bar{{h}_{c}}\left(\theta \right)/\tan \left(\theta \right)$.

The initial design steps share similarity to other conventional freeform eyepiece designs in ref. ^20^, aiming to achieve the correct optical power at the center field, satisfying structural constraints for manufacturability, and ensuring total internal reflection (TIR) for the first reflection within the main prism to ensure the correct ray propagation. Once a feasible starting point was identified, the design process departed from the conventional design methods by carefully managing the spatially varying optical power distribution to achieve the target angular resolution density distribution as depicted in Eq. (2), while optimizing the optical performance across the entire FOV. During the design process, we explored two different optimization strategies. In the first approach, during each optimization cycle, the actual chief ray heights at the micro-display panel, ${h}_{c}(\theta )$, were obtained through ray tracing across the FOV at the sampling interval $\Delta \theta$, which was set to 0.1% of the full FOV (about 0.1°). The local angular resolution density in PPD was estimated as:

4 $$F(\theta )=\frac{{h}_{c}(\theta +\Delta \theta )-{h}_{c}(\theta )}{{p}_{0}\times \Delta \theta }$$

At each iteration, the deviation of the resolution distribution of the current design from the target distribution specified in Eq. (2) was assessed and the optimization process iteratively minimized the difference. However, due to the freeform nature of the prism surfaces where local optical power cannot change rapidly and the limited number freedoms of the system, it is very challenging to strictly match the target resolution density distribution by strictly controlling the resolution distribution defined by Eq. (2) across the FOV, especially at the critical balance field points, such as the ${\theta }_{c1}$ and ${\theta }_{c2}$. A more robust approach is required to ensure smooth and continuous optical power variation, avoiding abrupt changes caused by localized surface shapes. Therefore, we developed an alternative strategy which controls the average resolution degradation rate, or equivalently the average optical power, over a region defined by the distance D on the display panel between the target field angle $\theta$ and the central field. This method ensures a gradual and natural transition in resolution across the display’s three functional regions, minimizing sudden variations. The distance D can be expressed as follows:

5 $$D(\theta )=\sqrt{(X(\theta )-X{(0))}^{2}+(Y(\theta )-Y{(0))}^{2}}$$

where the $X(\theta )$, $Y(\theta )$, $X(0)$, and $Y(0)$ are the X and Y local coordinates at the display panel plane for θ-angle and center field, respectively. By comparing it with the target image height distribution value $\bar{{h}_{c}}\left(\theta \right),$ we have effectively controlled the average degradation rate in resolution within the section across from the target field to the center field. The constraint for resolution distribution can be expressed as:

6 $$Metri{c}_{resolution\_distribution}=\left|\frac{{\sum }_{i}^{n}{D}_{i}(\theta )}{n}-{D}_{target}(\theta )\right|$$

where ${D}_{i}(\theta )$ is the distance at display panel from the center field to the field selected from one of the sampled fields with the angular distance *θ*, and the variable n denotes the total number of the fields with the same angular distance θ located at one field sampling ring. Another crucial aspect of optical performance is the control of rotational symmetry in the resolution distribution. This is achieved by controlling the variance of the distances among all the sampled fields with the same angular distance, defined as,

7 $$Metri{c}_{symmetry}={\sum }_{i}^{n}{[{D}_{i}(\theta )-\frac{{\sum }_{i}^{n}{D}_{i}(\theta )}{n}]}^{2}$$

In addition to satisfying TIR and structural constraints, we incorporate the metrics from Eqs. (6) and (7) to optimize the resolution distribution, aiming to align it with the target profile defined in Eq. (2) at selected field angles of 4°, 15°, and 30°. These angles are chosen based on two criteria: (1) the theoretical resolution function is relatively stable, and (2) they approximate the average resolution in the foveal, parafoveal, and peripheral regions, respectively. This selection supports a smooth, continuous transition in resolution distribution consistent with the desired statically foveated scheme. Ultimately, 60 variables were activated for optimization. The resolution distribution constraints from Eq. (6) were enforced within tolerances of 1 pixel at 4°, 8 pixels at 15°, and 50 pixels at 30°.

In the proposed static-foveation design, the desired angular resolution density is specified as a function of field angle. The MTF requirement is then defined with respect to the spatial frequency corresponding to the local angular resolution density, so the required MTF is naturally relaxed toward larger field angles as the target resolution decreases. During freeform optimization, we prioritize smooth, manufacturable spatially varying optical power while meeting the field-dependent MTF targets and practical constraints (e.g., prism geometry, TIR, and package thickness). Small deviations from the ideal target resolution distribution may therefore occur in transition regions, but the MTF remains sufficient at the corresponding local spatial frequencies.

### Simulated performance evaluation

**Fig. 6:**
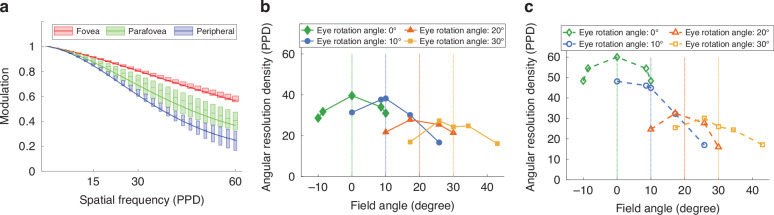
Simulated optical performance. **a** The MTF box plots of virtual image optical path of statically foveated freeform OST-HMD. **b** The perceived angular resolution density for four different angles of eye rotation at 0°, 10°, 20°, and 30°, respectively. **c** The angular resolution density of the Arizona eye model at four different angles of rotation at 0°, 10°, 20°, and 30°, respectively

To visualize the MTF performance of the freeform optics across different functional regions, Fig. 6a presents MTF box plots for a 4-mm eye pupil under green light illumination. The fovea, parafovea, and peripheral regions are shown in red, green, and blue, respectively. Each solid line indicates the average MTF value across six sampled fields within the corresponding region. The results indicate excellent performance in the foveal region, with average MTF values exceeding 60% at the spatial frequency of 60 PPD. The parafoveal region achieves an average MTF above 40% at 60 PPD, still providing a relatively high perceived resolution considering eye movement.

For a statically foveated display, it is critical to assess its perceived performance at different eye gaze angles. As shown in Fig. 2a, the perceived performance was evaluated by integrating the Arizona eye mode with the eyepiece. The display panel served as the object plane with uniform sampling of pixel locations, while the retina of the eye model acted as the image plane. The entrance pupil, located 3.05 mm behind the cornea, was aligned with the exit pupil of the foveated eyepiece. To evaluate the perceived performance at different eye gaze directions, the Arizona eye model was rotated around its center of rotation diagonally from 0° to 30° in 10° increments. For each rotation angle, MTF plots were generated, with tangential and radial values averaged. A 10% contrast modulation threshold at the cut-off frequency of 60 PPD was used as the perceived limiting resolution, which is consistent with the human visual system’s sensitivity to fine detail. The resulting angular resolution density as a function of field angle is shown in Fig. 6b for four different eye rotation angles. The peak perceived resolution was approximately 40 PPD in the fovea, 30 PPD in the parafovea, and 20 PPD in the periphery, indicating that the resolution degradation followed the expected trend. Within the typical range of eye movements, the perceived resolution degradation was minimal and imperceptible. The overall perceived resolution was lower than the designed eyepiece resolution due to the limited optical performance of the Arizona eye model, which drops rapidly at larger rotation angles [Fig. 6c]. Nevertheless, the system delivers high resolution in the fovea region where eye movements are most frequent, with controlled degradation in the periphery to reduce pixel demands while preserving peripheral vision.

## Data Availability

All data needed to evaluate the conclusions in the paper are present in the paper. Additional data related to this paper may be requested from the authors upon reasonable requests.
